# Physical Frailty and End-of-Life Care Small Groups

**DOI:** 10.5070/M5.53060

**Published:** 2026-10-31

**Authors:** Candace Leigh, Kevin Watkins

**Affiliations:** * Cleveland Clinic Akron General, Department of Emergency Medicine, Akron, OH

## Abstract

**Audience:**

This module was developed for emergency medicine (EM) residents, though may also be used for medical students on an emergency medicine clerkship or attending physicians.

**Length of Curriculum:**

This frailty and end-of-life care module consists of two 60-minute small group sessions.

**Introduction:**

Emergency medicine physicians often care for patients with significant comorbidities, physical frailty, and acute and chronic life-limiting conditions, including those at end-of-life. Over 60% of emergency department (ED) patients older than 65 years old meet established criteria for physical frailty, and at least half of older adults present to the ED in their final month of life.^1^,^2^ Therefore, it is critical that EM physicians understand end-of-life trajectories and adopt a thoughtful approach to end-of-life communication, clinical decision-making and symptom management to support both patients and their caregivers.^3^ To address this need, we developed a flipped-classroom case-based small group discussion session for emergency medicine learners that augments self-learning, followed by a small group discussion with peers.

**Educational Objectives:**

At the end of this module, learners should be able to: 1) recognize and classify end-of- life trajectories, 2) define physical frailty, including risk factors, prevalence and prognosis in ED patients, 3) develop a differential diagnosis and diagnostic and treatment plan for a patient with metastatic cancer presenting with fatigue, constipation, labile mood, and diffuse osseous pain, 4) review Medicare Hospice Benefit eligibility, 5) understand the differential diagnosis and medical management of chemotherapy-related nausea, 6) discuss the assessment and management of dyspnea in end-of-life patients, and 7) review how to estimate and communicate prognosis using practical timeframes.

**Educational Methods:**

This exercise consists of two 60-minute small group sessions (two clinical cases per session) using a “flipped classroom” design.^4^–^20^ The facilitator should distribute the learner responsible content one week in advance. Learners are instructed to complete the assigned readings and review four cases to prepare for an in-person discussion guided by the facilitator. Questions are included to guide their learning and structure the subsequent discussion. The facilitator uses the *Physical Frailty and End-of-Life Care Preceptor Notes*, which is organized around the handout questions, to stimulate discussion and answer questions.

**Research Methods:**

Learners evaluated the content using end-of-session anonymous feedback forms. Faculty small-group facilitators assessed learner participation.

**Results:**

We received open-ended responses from 11 learners. All felt the workload to prepare for the small-group session was reasonable and beneficial to their residency education. Some respondents requested additional sessions to discuss personal and/or difficult patient care experiences based on this “bite-sized” introduction to frailty and end-of-life care.

**Discussion:**

Overall, this split two-hour flipped classroom educational activity was an effective method to introduce the concept of frailty and end-of-life care to emergency medicine residents. Following the initial session, the authors added an additional case to expand the curriculum into two sessions to allow more time for free-form discussion.

**Topics:**

Emergency medicine, flipped classroom, medical education, teaching, end-of-life, frailty, hospice, palliative care, terminal dyspnea, malignant hypercalcemia, chemotherapy-induced nausea.

## USER GUIDE

**Table:** 

List of Resources:	
Abstract	24
User Guide	26
Small Groups Learning Materials	31
Appendix A: Small Group Application Exercise for Instructors	32
Appendix B: Small Group Application Exercise for Learners	58
Physical Frailty, Pallative and End-of-Life Pearls	71

**Learner Audience:**

This curriculum is designed for interns and junior and senior residents.

**Time Required for Implementation:**

The physical frailty, palliative, and end-of-life care module consists of two 60-minute small group sessions for a total of two hours of content. The pre-readings take learners between 30–60 minutes.

**Recommended Number of Learners Per Instructor:**

5–10

**Topics:**

Emergency medicine, flipped classroom, medical education, teaching, end-of-life, frailty, hospice, palliative care, terminal dyspnea, malignant hypercalcemia, chemotherapyinduced nausea.

**Objectives:**

After completing the frailty and end-of-life care module, learners should be able to understand the pathophysiology, management, and expected course for palliative and end-of-life emergencies, including:

1. End-of-Life Trajectories
  - List the four end-of-life trajectories
  - Describe the characteristics of these trajectories and list examples relevant to EM
2. Frailty
  - Define frailty and describe the incidence in ED patients
  - List possible reversible causes of decline in patients near end-of-life
  - Describe resources that may be available in the ED in managing patients with these conditions
  - Understand potential biases in treatment of these patients
3. Complications of Metastatic Malignancy
  - Discuss the differential diagnoses for decline in patients with metastatic malignancy
  - Discuss the diagnostic workup of these patients
  - Describe the pathophysiology, findings, and management of hypercalcemia
  - Understand the prognosis of patients with hypercalcemia in the setting of metastatic malignancy
4. Hospice
  - Describe eligibility for the Medicare Hospice Benefit
  - Review elements of discussion of hospice care with patients
5. Nausea in Malignancy
  - Understand the differential and medical management of chemotherapy-related nausea
6. Dyspnea at End-of-Life
  - List causes that contribute to dyspnea at end-of-life
  - Describe pharmacologic and non-pharmacologic therapies for dyspnea at end-of-life
  - Review the utility of an end-of-life dyspnea assessment tool
  - Describe the Respiratory Distress Observation Scale
  - Describe the Principle of Double Effect
7. Prognosis
  - Review the indicators that suggest a patient may be end-of-life
  - Discuss framing of the conversation around predicting the timeline of death

### Linked objectives and methods

These small group cases should have one to two faculty members or content experts as instructors with no more than 15 learners and 20 minutes per case. This allows for adequate discussion of both the developed cases and questions of real or plausible cases. Participants should be encouraged to share clinical experiences to enhance the discussion. Evaluations should be performed by learners on content and facilitators as well as faculty on resident participation. The addressed milestone of the end-of-life trajectories case is scientific knowledge (MK1). The addressed milestones of the frailty case are differential diagnosis (PC4) and scientific knowledge (MK1). The addressed milestones of the metastatic malignancy case are emergency stabilization (PC1), diagnostic studies (PC3), pharmacology (PC5), and scientific knowledge (MK1). The addressed milestone of the hospice case is system navigation (SBP3). The addressed milestones of the nausea in malignancy case are diagnosis (PC4), pharmacology (PC5), and scientific knowledge (MK1). The addressed milestones of the dyspnea at end-of-life case are diagnosis (PC4), pharmacology (PC5), and scientific knowledge (MK1). The addressed milestones of the prognosis case are system navigation (SBP3) and patient communication (ICS1).

### Recommended pre-reading for facilitator

- Bukovitz BJ. Top 7 things to know about frailty in the emergency department. *J Geriatr Emerg Med.* 2024; 4(5). DOI:https://doi.org/10.17294/2694-4715.1102
- Barker PC, Scherer JS. Illness trajectories: Description and clinical use. Palliative Care Network of Wisconsin. Published February 29, 2024. Accessed April 21, 2026. https://www.mypcnow.org/fast-fact/illnesstrajectories-description-and-clinical-use/
- Farkas J. Hypercalcemia. The Internet Book of Critical Care. Published November 20, 2025. Accessed April 19, 2026. https://emcrit.org/ibcc/hypercalcemia/
- Siddiqui F, Weissman D. Hypercalcemia of malignancy. Palliative Care Network of Wisconsin. Published July 9, 2024. Accessed April 19, 2026. https://www.mypcnow.org/fast-fact/hypercalcemia-of-malignancy/
- Lamba S, Quest TE, Weissman DE. Initiating a hospice referral from the emergency department. Palliative Care Network of Wisconsin. Published April 25, 2024. Accessed April 19, 2026. https://www.mypcnow.org/fast-fact/initiating-a-hospice-referral-from-the-emergency-department
- Magidson PD, Khoujah D, Anderson RS. Palliative care in the emergency department: Simplified symptomatic management. *J Geriatr Emerg Med*. 2021; 3(2).
- Murphy C, Simonis J. Assessment tools for dyspnea and respiratory distress at the end of life. Palliative Care Network of Wisconsin. Published November 29, 2022. Accessed April 19, 2026. https://www.mypcnow.org/fast-fact/assessmenttools-for-dyspnea-and-respiratory-distress-at-the-end-of-life/
- Selman K, Shenvi C. What’s in a name? Understanding failure to thrive and frailty in the emergency department. *J Geriatr Emerg Med.* 2022; 1(3).
- Wolf LA, Lo AX, Serina P, et al. Frailty assessment tools in the emergency department: A geriatric emergency department guidelines 2.0 scoping review. *J Am Coll Emerg Physicians Open*. 2023;5(1):e13084. Published 2023 Dec 29. doi:10.1002/emp2.13084
- Siegel M, Bigelow S. Palliative care symptom management in the emergency department: The ABC's of symptom management for the emergency physician. *J Emerg Med*. 2018;54(1):25–32. doi:10.1016/j.jemermed.2017.08.004
- Farkas J. Nausea, emesis, and antiemetics. Internet Book of Critical Care. Published April 8, 2025. Accessed May 6, 2026. https://emcrit.org/ibcc/antiemetic/

### Optional further reading for facilitator

- Smith AK, McCarthy E, Weber E, et al. Half of older Americans seen in emergency department in last month of life; most admitted to hospital, and many die there. *Health Aff* (Millwood). 2012; 31(6):1277–85. doi:10.1377/hlthaff.2011.0922
- Shoenberger J, Lamba S, Goett R, et al. Development of hospice and palliative medicine knowledge and skills for emergency medicine residents: Using the Accreditation Council for Graduate Medical Education Milestone Framework. *AEM Educ Train*. 2018;2(2):130–145. Published 2018 Mar 22. doi:10.1002/aet2.10088
- Xue QL. The frailty syndrome: Definition and natural history. *Clin Geriatr Med*. 2011;27(1):1–15. doi:10.1016/j.cger.2010.08.009
- Martin FC, Ranhoff AH. Frailty and sarcopenia. 2020 Aug 21. In: Falaschi P, Marsh D, eds. *Orthogeriatrics: The Management of Older Patients with Fragility Fractures*. 2nd ed. Cham (CH): Springer; 2021. Chapter 4. Available from: https://www.ncbi.nlm.nih.gov/books/NBK565582/

### Learner responsible content (LRC)

- Bukovitz, Brandon J. Top 7 things to know about frailty in the emergency department. *J Geriatr Emerg Med.* 2024; 4(5). doi:https://doi.org/10.17294/2694-4715.1102
- Barker PC, Scherer JS. Illness trajectories: Description and clinical use. Palliative Care Network of Wisconsin. Published February 29, 2024. Accessed April 21, 2026. https://www.mypcnow.org/fast-fact/illnesstrajectories-description-and-clinical-use/
- Farkas, J. *Hypercalcemia.* The Internet Book of Critical Care. Published November 20, 2025. Accessed April 19, 2026. https://emcrit.org/ibcc/hypercalcemia/
- Burns E, Buttner R. *Hypercalcaemia.* Life in the Fast Lane. Published October 8, 2024. Accessed April 19, 2026. https://litfl.com/hypercalcaemia-ecg-library/
- Siddiqui F, Weissman D. Hypercalcemia of malignancy. Palliative Care Network of Wisconsin. Published July 9, 2024. Accessed April 19, 2026. https://www.mypcnow.org/fast-fact/hypercalcemia-of-malignancy/
- Lamba S, Quest TE, Weissman DE. Initiating a hospice referral from the emergency department. Palliative Care Network of Wisconsin. Published April 25, 2024. Accessed April 19, 2026. https://www.mypcnow.org/fast-fact/initiating-a-hospice-referral-from-the-emergency-department
- Magidson PD, Khoujah D, Anderson RS. Palliative care in the emergency department: Simplified symptomatic management. *J Geriatr Emerg Med*. 2021; 3(2).
- Murphy C, Simonis J. *Assessment Tools for Dyspnea and Respiratory Distress at the End of Life.* Palliative Care Network of Wisconsin Published November 29, 2022. Accessed April 19, 2026. https://www.mypcnow.org/fast-fact/assessmenttools-for-dyspnea-and-respiratory-distress-at-the-end-of-life/
- Zhuang Q, Yang GM, Neo SH, Cheung YB. Validity, reliability, and diagnostic accuracy of the Respiratory Distress Observation Scale for assessment of dyspnea in adult palliative care patients. *J Pain Symptom Manag*. 2019;57(2):304–310. doi:10.1016/j.jpainsymman.2018.10.506
- Farkas J. Nausea, emesis, and antiemetics. Internet Book of Critical Care. Published April 8, 2025. Accessed May 6, 2026 .https://emcrit.org/ibcc/antiemetic/

### Small group application exercise (sGAE)

See the following attached materials for this small group exercise

- Appendix A: Small Group Application Exercise for Instructors
- Appendix B: Small Group Application Exercise for Learners
- Physical Frailty, Pallative and End-of-Life Pearls

### Results and Tips for Successful Implementation

This flipped classroom session is best implemented in small groups (5–10 learners) with a senior resident or attending physician leading as a facilitator. Additional individuals present with specialized training or interest in frailty or end-of-life care is helpful but not required. This session was presented at didactics at a 3-year EM program comprised of 33 residents.

We measured outcomes with an anonymous survey sent to EM residents in attendance that consisted of three open-ended questions after listing their postgraduate year: How was the workload to prepare for this small group? How was the content of this small group, and was it appropriate/beneficial as an ED resident? What was your general impression of this small group? Any other feedback?

Eleven residents completed this survey (11/33 total, or 33% response rate), with most being senior residents (46% PGY3, 27% PGY2 and 27% PGY1). All participants reported that the time required for pre-session reading and small group preparation was reasonable in scope, the content was clinically relevant, and it added an important dimension to their residency education. Some respondents requested additional sessions to discuss applying this information to personal and/or difficult patient care experiences based on this “bite-sized” introduction to frailty, palliative and end-of-life care.

## Figures and Tables

**Figure:**
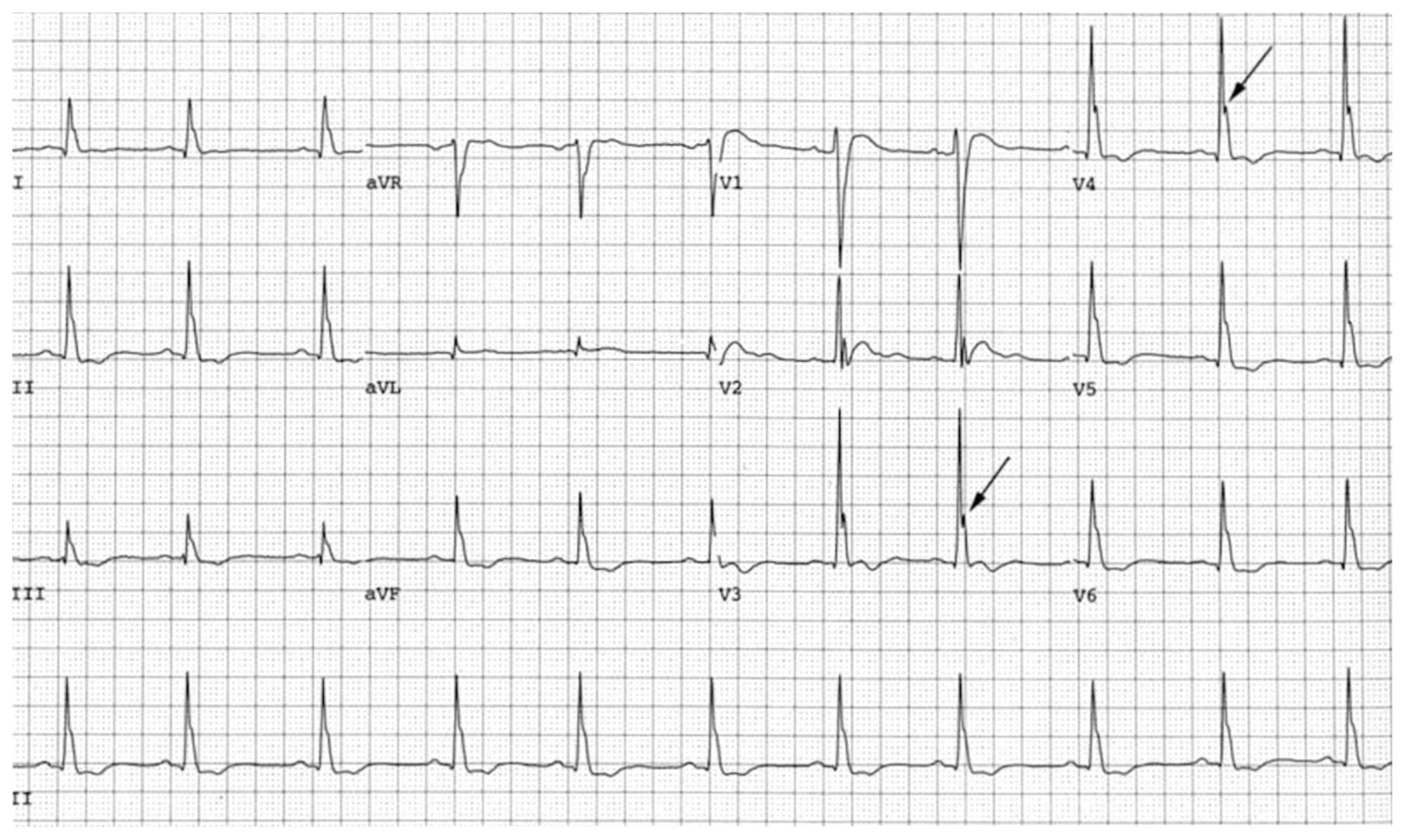
QRS notching (Osborne Waves) in hypercalcemia.^33^

**Figure:**
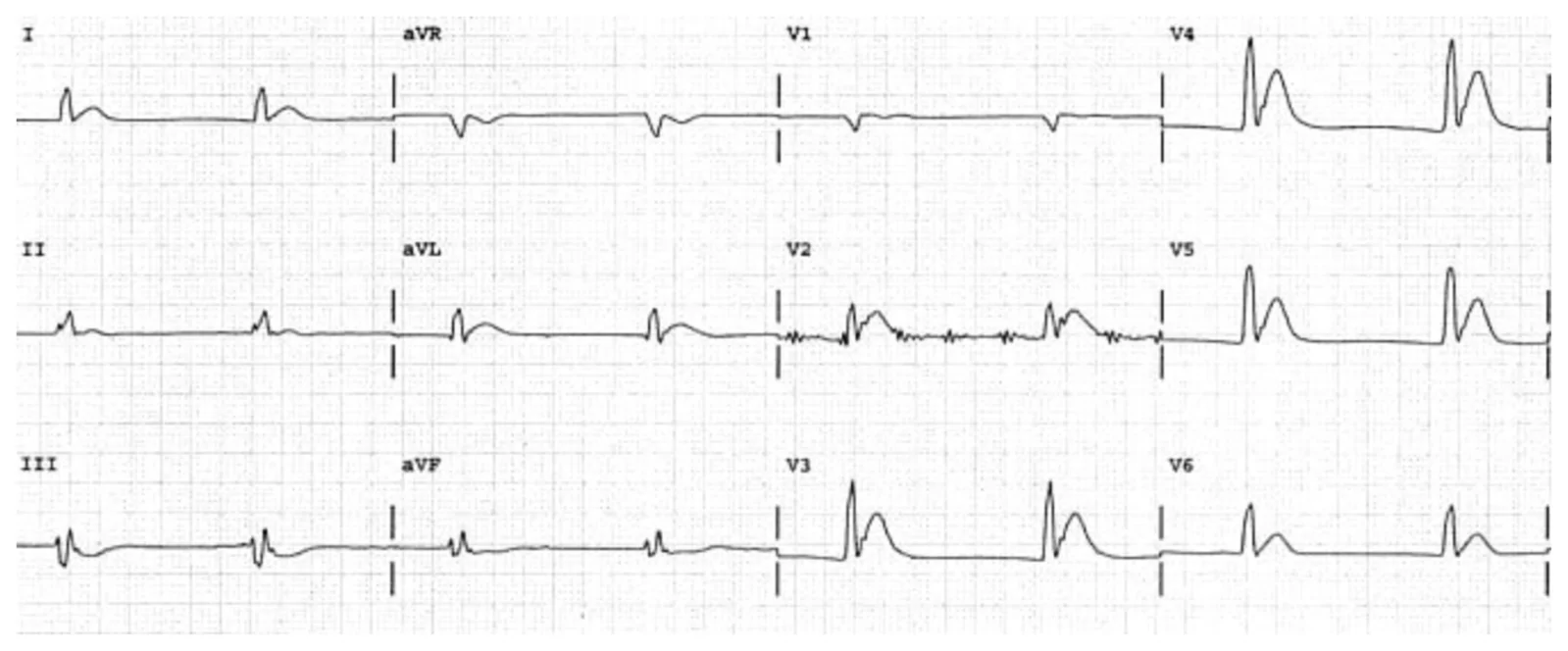
QT segment shortening in hypercalcemia.^33^

## SMALL GROUPS LEARNING MATERIALS

### Small Group Application Exercise (sGAE)

**Stations:** This exercise is best deployed in small groups (5–10 learners of a variety of training levels from medical students to senior residents) with a senior resident as the group leader. Suggested reading and patient cases with questions for learners are sent out one week in advance. This is designed for two 60-minute small group sessions (two cases each) using a “flipped classroom” design.

## Appendix A Small Group Application Exercise for Instructors

### Physical Frailty, Palliative and End-of-Life Care Case Studies

**Case 1:**

Mr. Michaels is an 84-year-old male presenting from home with a chief complaint of “fall.” He is moved from the EMS cot to your last monitored ED room. His daughter Jane rushes to his bedside, insistent that she provide his history, stating, “He is forgetful.” He is pleasant but overwhelmed by all the commotion in the room. He denies any pain, shortness of breath, headache, neck or back pain, nausea or vomiting. He cannot recall events that occurred prior to arrival. He only asks for his glasses, which seem to be missing. Jane fills in the details leading to the ED presentation:

“I was getting him up for his morning routine of breakfast and coffee. He went to pivot from the bed to his walker, when he must have lost his balance and fell, striking his head against the nightstand. I tried multiple times to get him up, but he was just too weak. I called the paramedics, and once they saw the bruise on his head and the wounds on his arms, they urged us to come in to be seen. This is his third visit to the ED in the last six weeks despite a recent hospital and rehabilitation stay. I moved him to stay with me in our spare room so I can look after him. We are doing everything the physical therapist told us, and he is still falling and barely eating. I doubt he is sleeping - neither of us are. He is restless and wants to roam the house all night. I’m not sure what more I can do. I am exhausted.”

Past Medical History

1. Parkinson’s disease
2. Benign prostatic hypertrophy
3. Vision and hearing impairment
4. Diet-controlled type 2 diabetes mellitus
5. Hypertension
6. Hyperlipidemia

Medications

1. Carbidopa-levodopa 25–100 mg PO three times daily
2. Quetiapine 50 mg PO at bedtime
3. Tamsulosin 0.4 mg PO daily
4. Atorvastatin 10 mg PO daily
5. Aspirin 81 mg PO daily

At Triage:

- Temperature (T) 98.0°F
- Heart rate (HR) 62 beats/min
- Blood pressure (BP) 106/78 mmHg
- Respiratory rate (RR) 18/min
- Oxygen saturation (O_2_sat) 97% on room air

Physical exam: The patient is alert and oriented to self and place and very cachectic; however, he appears to be in no distress. He has an abrasion to his left frontal scalp and multiple new skin tears to his upper and lower extremities. He also has a stage 1 sacral decubitus ulcer, new from the last ED visit 1 week ago. His chest, abdomen, back and neck are atraumatic. He has slight cogwheel rigidity and a resting tremor.

**Question Prompts:**

1. List the four global end-of-life trajectories, their characteristics, and include two ED patient examples of each.
  - Terminal Illness – A disease trajectory that is long, then punctuated by a shorter phase of sharp decline over weeks to months.^21^ Examples – malignancy, multiple sclerosis, HIV/AIDS.
  - Organ Failure – A slow steady decline in physical function, involving intermittent severe symptom crises, where recovery rarely reaches a patient’s level of pre-crisis function.^21^ Examples – heart failure, COPD, liver disease.
  - Frailty – A very slow decline in function, often associated with dementia, post-stroke or progressive degenerative disease. Patients often die from sepsis, renal failure or another illness.^21^ Examples - Alzheimer’s dementia, Parkinson’s disease, advanced cerebrovascular disease.
  - Sudden Death – Abrupt, no preceding decline.^21^ Examples – myocardial infarction resulting in cardiac arrest, intracranial hemorrhage, severe trauma (only about 10–15% of deaths are due to the Sudden Death trajectory).^25^
2. Draw graphs representing each of the four trajectories (patient function on Y axis and time on X axis).^21^
3. What is physical frailty, and how prevalent is it among emergency department patients?
  Physical frailty is defined as three of the five following: slow walking speed, fatigue, low physical activity, weight loss, weakness. It is associated with dysregulation of multiple physiologic stress responses including the immune, endocrine, inflammatory and metabolic systems, rendering patients to be at high risk for falls, depression, incontinence, disability and cognitive impairment.^1^ Frailty is a risk factor for severe illness and a prognostic marker for future clinical outcomes. The incidence can be as high as 62% in emergency department patients over 65 years of age.^1^
  Discussion Points: “Frailty” vs “Failure to Thrive” – while one may see these terms interchangeable in general practice or literature, there’s a push to further define and differentiate between the two. “Failure to Thrive (FTT)” is a term that derived from pediatrics, a diagnosis given to children when they are not meeting growth or developmental expectations.^22^ In adults, this term is often used in patients (geriatric age or not) when presenting with nonspecific or vague symptoms such as weakness, fatigue, confusion, weight loss, and overall functional decline. This is problematic because it can create bias in physicians to assume these symptoms are chronic or age-related, potentially missing emergency conditions with atypical symptoms, effects of polypharmacy or other social and behavioral health causes. One study demonstrated that 88% of patients with a diagnosis of FTT had acute medical problems, mostly infections, requiring longer and more complex hospitalizations compared with those simply needing facility placement.^26^ In contrast, “frailty” is a measurable, geriatric syndrome that aids in assessing function, risk and appropriate disposition.^27^
4. What indicators (functional changes, symptoms) suggest that a patient is nearing end-of-life (final months of life)?
  - Extreme frailty and dependent activities of daily living (ADLs) including frequent falls, impaired balance, bedbound status, and pressure wounds.^28^
  - Weight loss/sarcopenia.^28^
  - Refractory dyspnea or chest pain at rest. 29
  - Recurrent infections (aspiration pneumonia, urinary tract infection).^29^
    Discussion points: These indicators provide an overall assessment of prognosis. ED physicians face time, environmental and other situational barriers that make discussing prognosis related to physical frailty challenging. However, we also have the benefit of meeting a patient for the first time and evaluating them through an objective lens. It is especially important to consider discussing frailty and prognosis in the patient that has a high likelihood of requiring acute high-risk interventions (surgery, procedures) or medical life-supports (dialysis). Reluctance to initiate these discussions can lead to poor symptom management (if emphasis is on changing disease trajectory over addressing symptoms), mismatched expectations (unrealistic optimism), or a family unprepared for a patient’s decline or death. The Clinical Frailty Score (CFS) is an easy tool to measure frailty that can predict in-hospital and 30-day mortality (CFS ≥ 5), and at the very least may help ED clinicians decipher which patients are good candidates for an inpatient or outpatient geriatric assessment or in need of further support at home because at least 20% of frail adults return to the ED within 30 days of discharge. 2,30 Frailty care in the ED is still developing, including how frailty screening can aid in improving patient outcomes.^27^
    **Table:** The Clinical Frailty Score (CFS)^31^
    1. Very fit	People who are robust, active, energetic, and motivated. These people commonly exercise regularly. They are among the fittest for their age.
    2. Well	People who have no active disease symptoms but are less fit than people in category 1. Often, they exercise or are very active occasionally, eg, seasonally.
    3. Managing well	People whose medical problems are well controlled but are not regularly active beyond routine walking.
    4. Vulnerable	While not dependent on others for daily help, often symptoms limit activities. A common complaint is being “slowed up,” and/or being tired during the day.
    5. Mildly frail	These people often have more evident slowing, and need help in high order IADLs (instrumental activities of daily living) such as finances, transportation, heavy housework, and medications. Typically, mild frailty progressively impairs shopping and walking outside alone, meal preparation and housework.
    6. Moderately frail	People need help with all outside activities and with keeping house. Inside, they often have problems with stairs and need help with bathing and might need minimal assistance (cuing, standby) with dressing.
    7. Severely frail	Completely dependent for personal care, from whatever cause (physical or cognitive). Even so, they seem stable and not at high risk of dying (within ~ 6 months).
    8. Very severely frail	Completely dependent, approaching the end of life. Typically, they could not recover even from a minor illness.
    9. Terminally ill	Approaching the end of life. This category applies to people with a life expectancy <6 months, who are not otherwise evidently frail.
5. What are the potential reversible causes of Mr. Michael’s functional decline?
  - Delirium secondary to infection or other acute condition (urinary retention).^22^
  - Polytrauma.^22^
  - Polypharmacy.^22^
  - Alternative long-term diagnosis.^22^
  - Depression or behavioral health disturbance.^22^
  - Sleep-wake disturbances.^22^
  - Environmental or home-related barriers.^22^
  - Caregiver fatigue, neglect or abuse.^22^
  - What resources may you have in the emergency department to assist Mr. Michaels and his daughter?
    Social work, case management, caregiver support services, geriatric or palliative specialists, primary care team. When caring for these patients in low-resource emergency care settings, it may be helpful to refer patients and their caregivers to The National Institute on Aging for resources on finding long-term or home health care, and nutrition and activity recommendations: www.nia.nih.gov
6. What biases or patient history features might influence how clinicians perceive or treat patients like Mr. Michaels?
  - Ageism^22^
  - Assumptions about quality of life^22^
  - Perceived urgency based on triage chief complaint (“failure to thrive”)^22^
  - Patient’s history of prior advanced care planning documentation such as a current do-not-resuscitate order^22^
  - Dependency on long term care^22^
  - Patients’ lack of capacity to make their own healthcare decisions^22^
  - ED physician’s own personal opinions about end-of-life and quality of life^22^

Suggested resources for learners:

Bukovitz, BJ. Top 7 things to know about frailty in the emergency department. *J Geriatr Emerg Med.* 2024; 4(5). DOI: https://doi.org/10.17294/2694-4715.1102

Barker PC, Scherer JS. Illness trajectories: Description and clinical use. Palliative Care Network of Wisconsin. Published February 29, 2024. Accessed April 21, 2026. https://www.mypcnow.org/fast-fact/illness-trajectories-description-and-clinical-use/

**Case 2:**

Mrs. Green is a 50-year-old female presenting with progressively worsening fatigue, nausea, severe thirst and diffuse osseous pain since receiving her last session of palliative chemotherapy four weeks ago. She also describes having severe constipation (last bowel movement was “about a week ago”), a labile and irritable mood, anorexia, and headaches, but denies fever, falls/trauma, or ill contacts.

Past Medical History

1. Breast adenocarcinoma, metastatic to the lung, liver, bone
2. Peptic ulcer disease

Medications

1. Parenteral chemotherapy q2 weeks (recently discontinued due to disease progression)
2. Omeprazole 40 mg PO daily
3. Ondansetron 8 mg PO every 8 hours PRN nausea
4. Oxycodone 5–10 mg PO every 6 hours PRN pain
5. Dexamethasone 2 mg PO twice daily
6. Antacids PRN

In Triage:

- Temperature (T) 98.8°F
- Heart rate (HR) 114 beats/min
- Blood pressure (BP) 102/67 mmHg
- Respiratory rate (RR) 22/min
- Oxygen saturation (O_2_sat) 96% on room air

Physical exam: The patient is awake and alert, answering questions and following commands. Her mucous membranes are dry. Her HR is elevated without audible murmurs. She has mild abdominal tenderness diffusely. Palpation of her extremities, especially her hips and lower legs cause her to grimace in pain. There are no wounds or rashes.

**Question Prompts:**

1. What is your differential diagnosis?
  DDX –acute kidney injury, hypothyroidism, hypercalcemia of malignancy, opioid induced constipation, milk-alkali syndrome, infection, hyperglycemia, intracranial metastasis
2. What ED testing would you recommend? What do you expect these diagnostic studies to reveal?
  - EKG – normal or QT shortening. With a very short QT, one can mistake the takeoff of the T wave for an elevated ST segment. It can also cause notching of the end of the QRS segment (Osborne waves), or AV block at extremely high levels.
  - Venous Blood Gas for serum pH
  - CBC
  - CMP – evaluate for acute kidney injury (AKI)
  - Ionized calcium (iCa) – moderately high levels expected in this patient – total calcium 12–14 mg/dl, ionized Ca 3–3.5 mm. While a total calcium level can be a helpful screening test, ionized calcium measures biologically active calcium. Evidence suggests that formulas that exist to calculate a correct calcium level with respect to albumin are relatively inaccurate.
  - Magnesium
  - Phosphorus – hypophosphatemia suggests hyperparathyroidism, humeral hypercalcemia or sometimes milk-alkali syndrome.
  - TSH and free T4
  - UA – assess for infection, dehydration.
  - POCUS – assists in assessment of hydration status.
  - Consider parathyroid hormone (PTH) or PTH-related peptide levels, 25-OH and 1,25-OH vitamin D levels.
    Discussion: Laboratory ionized calcium can influence severity and types of symptoms.^32^
    - Normal levels: 8.5–10.5 mg/dL (iCa 1.12–1.32 mm) – asymptomatic
    - Mild hyperCa: 10.5–11.9 mg/dL (iCa 1.32–3 mm) – asymptomatic or mild symptoms
    - Moderate hyperCa: 12–13.9 mg/dL (iCa 3–3.5 mm) – symptomatic.
    - Severe hyperCa: >14 mg/dL (iCa >3.5 mm) - coma, bradycardia, AV block.
3. Describe the pathophysiology of this patient’s condition as well as other etiologies of this condition.
  - Hypercalcemia of malignancy
    - Humoral causes (80%) - PTH-related protein secretion by some tumors (neuroendocrine), typically due to squamous cell, urothelial, breast, endometrial, ovarian, pancreatic malignancies or lymphoma.^32^
    - Osteolytic causes (20%) – osseous metastasis due to breast, lung, prostate malignancy, myeloma, leukemia or lymphomas.^32^
    - Other - 1–25(OH)2D (vitamin D) secreting lymphomas, ectopic hyperparathyroidism.
  - Other hyperparathyroidism – adenoma, parathyroid hyperplasia, ESRD.^32^
  - Medications
    - Excess vitamin A or D^32^
    - Milk alkali syndrome – dietary Ca intake, antacids^32^
    - Thiazides – increase Ca reabsorption in kidney^32^
    - Lithium – interferes with calcium-sensing receptor in parathyroid gland^32^
    - Theophylline – enhances effects of parathyroid hormone^32^
    - Total Parenteral Nutrition (TPN) with excessive Ca^32^
    - Rebound effects from discontinuing certain medications – denosumab (used for osteoporosis and for fracture prevention in individuals with osseous malignancy).^32^
  - Autoimmune granulomatous conditions – sarcoid, inflammatory bowel^32^
  - Infections – tuberculosis and some fungal infections^32^
4. What are the classic presenting symptoms and physical exam findings of this condition?
  - “Stones, bones, [abdominal] groans, psychiatric overtones.^32^
  - Neurologic – fatigue, delirium, coma, hyporeflexia.^32^
  - Gastrointestinal – decreased PO intake, nausea, vomiting, constipation, pancreatitis.^32^
  - Renal – dehydration, polyuria/polydipsia, renal stones, acute kidney injury.^32^
  - Musculoskeletal – weakness, bone pain, pathologic fractures.^32^
  - Cardiovascular – bradycardia, hypertension.^32^
5. What urgent treatments are indicated in the emergency department for this patient?
  - Intravenous Hydration – administered to support renal function and diuresis, functionally facilitating the excretion of Ca. Most patients with moderate to severe hyperCa are severely volume depleted, so the goal of resuscitation is to move to an euvolemic state. It is important to remember that lactated ringers contain Ca and should be avoided.^32^
  - Loop diuretics (furosemide) – parenteral, works to inhibit absorption of Ca, though it should be given after appropriate hydration. This therapy may not be as effective as previously thought.^32^
  - Stop any contributing medications^32^
  - Calcitonin - inhibits osteoclast activity in bone and promotes urinary Ca excretion. The dose is 4 units/kg either SQ or IM, though the IM route is uncomfortable for patients. Common medication side effects are nausea, vomiting, flushing and pain at site. This therapy is reserved for those with moderate to severe hyperCa.^32^
  - Intravenous bisphosphonates (zoledronic acid) – stops Ca resorption into the serum from the bone by causing osteoclast apoptosis. Caution with renal failure and in premenopausal women (drug can remain in bone and be released during pregnancy). This drug is not to be used with hyperCa due to milk-alkali syndrome. The dose of zoledronic acid is 4mg IV. Benefits cannot be seen for a few days; therefore. starting early in course is advised, although it may be helpful to use in consultation with a subspecialist (oncology or endocrinology). Common side effects include flu-like symptoms. Monitor closely for hypoCa, hypophosphatemia, or renal failure during use. This therapy is also reserved for those with moderate to severe hyperCa.^32^
  - Refractory cases – denosumab, glucocorticoids, dialysis. 32
6. What is an appropriate disposition for this patient?
  Individuals with mild hypocalcemia (mostly asymptomatic or with mild symptoms, Ca 10.5–11.9mg/dL or iCa 1.32–3 mm) can often be discharged home with close follow-up. With this patient’s symptoms – her levels are likely in the moderate range and inpatient monitoring and treatments are indicated.^32^
7. What is the prognosis for this condition?
  Half of patients with hypercalcemia of malignancy die within 30 days, even if hypercalcemia is corrected.^34^
8. You read in the electronic medical record that the patient’s oncology team is no longer recommending palliative chemotherapy and recently offered to connect her with a local hospice agency. Is Mrs. Green eligible to invoke the Medicare Hospice Benefit?
  Yes, Mrs. Green has a prognosis of six months or less if her disease runs its expected course; therefore, she is eligible for hospice services.^23^,^34^
9. What advice would you give her at this point in her illness? How do you describe hospice care to your ED patients?
  - Start with a discussion about what she knows about her disease – “What have you been told about your cancer?”^23^
  - Survey her knowledge, what does she remember about her recent discussion with her oncologist, “Has anyone discussed hospice with you? What do you remember from that discussion?”^23^
  - If her goal is to avoid hospitalization, offer to connect the patient with a hospice agency. Hospice care can provide 24/7 on-call assistance, medications and visits for symptom management, education, emotional and spiritual care support. This care can occur in the home, a nursing facility or in an inpatient hospice care center, and can be often initiated from the ED. A hospice agency will need to know the hospice-eligible diagnosis, which physician will be following on hospice (in most cases from ED referrals, this may be the patient’s primary care physician, oncologist or the hospice agency’s medical director), and what necessary equipment is needed immediately (oxygen, hospital bed, etc). In many cases, hospice enrollment can often be arranged in a patient’s home within 24–48 hours if discharged from the ED. In this case, the hospice agency may ask the ED physician to write medication prescriptions for pain, dyspnea, nausea, to support patients until enrollment can be completed.^23^

Suggested resources for learners:

Farkas, J. Hypercalcemia. The Internet Book of Critical Care. Published November 20, 2025. Accessed April 19, 2026. https://emcrit.org/ibcc/hypercalcemia/

Burns E, Buttner R. Hypercalcaemia. Life in the Fast Lane. Published October 8, 2024. Accessed April 19, 2026. https://litfl.com/hypercalcaemia-ecg-library/

Siddiqui F, Weissman D. Hypercalcemia of malignancy. Palliative Care Network of Wisconsin. Published July 9, 2024. Accessed April 19, 2026. https://www.mypcnow.org/fastfact/hypercalcemia-of-malignancy/

Lamba S, Quest TE, Weissman DE. Initiating a hospice referral from the emergency department. Palliative Care Network of Wisconsin. Published April 25, 2024. Accessed April 19, 2026. https://www.mypcnow.org/fast-fact/initiating-a-hospice-referral-from-the-emergencydepartment

**Case 3:**

Mr. Jones is a 39-year-old male presenting with persistent non-bloody emesis for the past three days after his chemotherapy infusion (FOLFIRINOX). He notes that for the last 24 hours, despite taking in small sips of oral fluids and ondansetron (8mg PO every 6 hours), everything he eats or drinks results in emesis. Just in the past few hours, he has been experiencing acute worsening of his cancer-related epigastric abdominal pain and new lightheadedness with standing. He denies fever, chills, dyspnea or chest pain. His last bowel movement was a few hours ago and consisted of diarrhea, which is typical for him in the days following his infusions. The last time this happened he required a prolonged hospitalization.

Past Medical History

1. Metastatic pancreatic adenocarcinoma
2. GERD
3. Opioid use disorder in recovery

Medications

1. Omeprazole 20mg PO daily
2. Olanzapine 10mg PO QHS
3. Methadone 80mg PO daily
4. Oxycodone IR 10–20mg PO every 4 hours PRN
5. Multivitamin
6. Pancreatic Enzymes

Allergies

None

Vital Signs:

- Temperature (T) 37.7°C
- Blood Pressure (BP) 101/65 mmHg
- Heart Rate (HR) 114 beats/min
- Respiratory Rate (RR) 20/min
- Oxygen Saturation (O_2_sat) 95% on room air
- Weight 60kg

Physical exam: The patient has cachexia, dry mucous membranes, epigastric tenderness with minimal voluntary guarding, and no distention. The HR is elevated but regular. He has a normal mental status without focal neurological deficits, and a slow but steady gait.

Laboratory studies are pending.

The EKG is notable for sinus tachycardia with a corrected QT interval (QTc) of 480 milliseconds (ms).

**Question prompts:**

1. In a general approach to emergency department patients with advanced malignancy presenting with nausea and vomiting, name at least five differential diagnoses:
  - Medication-related side effect or withdrawal (chemotherapy, opioids, antibiotics)^35^,^36^
  - Bowel/gastric outlet obstruction^35^,^36^
  - Cholangitis/biliary obstruction^35^,^36^
  - Pancreatitis^35^,^36^
  - Constipation (malignancy or opioid use related)^35^,^36^
  - Gastroparesis/Ileus (electrolyte disturbance)^35^,^36^
  - High intracranial pressure related to CNS metastasis/malignancy^35^,^36^
  - Uremia^35^,^36^
  - Hypercalcemia of malignancy^35^,^36^
2. What components of the physical exam are the most pertinent to explore in this population?
  - Abdominal exam: bowel sounds, tenderness, mass, distention or fluid wave^35^
  - Neurologic exam: assess for focal deficits, posterior circulation findings^35^
  - Volume status: capillary refill, mucous membranes, skin turgor^35^
  - Skin changes: jaundice, bruising^35^
3. What laboratory and/or imaging studies are appropriate to order:
  - Chemistries (renal panel, including Ca, Mg, Phos)^35^
  - Liver function tests^35^
  - CBC (assess for pancytopenia in setting of chemotherapy)^35^
  - Lipase^35^
  - Urinalysis^35^
  - Pregnancy test (when appropriate)^35^
  - Lactate (if infection or ischemia is considered)^35^
  - Imaging Central Nervous System (CNS), abdominal^35^
4. What are the difficulties in treating this patient’s nausea?
  - Prolonged QT interval: related to electrolyte disturbance or methadone side effect, limits possible antiemetic options.^36^
  - Olanzapine ineffectiveness^36^
  - Chronic pain^36^
5. 5. Fill out the following table:
  **Table:**
  Antiemetics (modified by authors)^35^,^36^					
  Drug	Dose	Receptor	QT	Side effects	Comments
  Ondansetron	4mg or 8mg IV/PO	5-HT3	(+++)	constipation, headache	First line for chemo-induced nausea, moderate QT risk, especially with IV/high dose (8–16mg IV) or with concurrent electrolyte disturbance, rare serotonergic interaction risk
  Droperidol*	0.625–2.5mg IV/IM	central D2/H1	(+++)	EPS, neuroleptic malignant syndrome, sedation	Especially helpful in treating cannabinoid hyperemesis syndrome, droperidol half-life is 2–3 hours Black Box Warning for QT prolongation - can occur at therapeutic doses
  Haloperidol Lactate*	1–5mg IV/IM	central D2/H1	(++)	QT prolongation, Torsades	Haloperidol half-life 14–26 IV, up to 37 hours PO
  Prochlorperazine*	5–10mg PO/IM/IV	D2 primarily	very mild risk	EPS, sedation	Useful for migraine HA, mostly anti-D2 selective
  Promethazine*	12.5–25mg PO/IM/PR	Mostly H1	(+)	Sedation	Phenergan has risk of tissue necrosis with IV extravasation, avoid IV use unless through central access or significantly diluted
  Olanzapine*	5–10mg PO/ODT , 2.5mg IM	D2/5HT3/H1	very mild risk	EPS, sedation, orthostatic hypotension	May lower seizure threshold; you may see this added by oncology for appetite benefits, broad spectrum D2 and 5HT3 effects
  Metoclopramide*	10–20mg IV/PO	D2/5HT3	(+)	EPS, diarrhea	Prokinetic agent, so avoid in suspected bowel obstruction or opioid use constipation, useful in gastroparesis, weak antiemetic
  Diphenhydramine	25mg IV/PO	H1	none	sedation	May increase QT prolonging effect of QT prolonging agents
  Lorazepam	0.5mgIV/PO	GABA	none	sedation	Weak antiemetic, may be useful in anticipatory nausea or anxiety
  Dexamethasone	4–8mgIV/PO		none	mood effects, elevated glucose	Consult with oncologist in patients on immunotherapy regimens since steroid may interfere with treatment; especially useful in nausea related to CNS malignancy or bowel edema, can be pain adjuvant as well
  Isopropyl alcohol	sniff		none		Evidence based adjunct, rapid, safe
  H1=histamine type 1; GABA = gamma-aminobutryic acid; D2= dopamine type 2; 5-HT3 = serotonin type 3
  EPS = extrapyramidal symptoms (tardive dyskinesia, akathisia, dyskinesia, dystonia, oculogyric crises)
  * Use caution or avoid dopamine antagonists in Parkinson's disease due to worsening motor symptoms
6. What would be your next step in treating this patient’s nausea and why?
  - IV hydration^35^
    - Electrolyte correction (K>4, Mag>2)^35^
    - Assess for obstruction, CNS process, pancreatitis, hypercalcemia, or other etiologies^35^
    - In patients with prolonged QT, use the lowest-risk agents available (olanzapine, lorazepam, dexamethasone, prochlorperazine, low dose haloperidol or droperidol, isopropyl alcohol), while avoiding serial stacking of QT prolonging agents whenever possible. QTc monitoring is suggested regardless of choice in therapy. Note that this patient is already on nightly olanzapine. It’s best to first check for adherence and follow with a breakthrough dose. Anticipating and treating electrolyte abnormalities will help prevent QT-related complications in this patient (IV hydration-related dilution).^35^
    - Adequate pain control may also improve nausea. Most effective agents for pain will depend on the components of pain, opioid tolerance and allergies. Methadone withdrawal may also be a possible contributor – however, the half-life is very long and variable, 8–59 hours).^37^ So asking when he has last been able to tolerate his methadone dose is important. If he is still unable to tolerate methadone orally, consult with your pharmacist, oncologist or palliative care consultants to discuss parenteral methadone bridging.
      Discussion Points:
      - Olanzapine is a highly underutilized antiemetic in the emergency department, likely due to its sedating effects, long half-life and its association to psychiatric treatment.^35^ Many oncology patients may already be prescribed a single nightly dose of olanzapine (2–10mg) taken before bedtime rather than as an as-needed antiemetic.^38^ If considering prescribing low dose olanzapine at ED discharge, notify the patient of alternative uses since it may come up in discussions with the retail pharmacist or in the medication package insert.
      - While not discussed above, another class of antiemetic medications is the NK1 antagonists (eg, aprepitant/fosaprepitant). These agents do not prolong the QT interval and are highly effective in the prevention of chemotherapy-induced nausea and vomiting. However, they are not typically used as rescue medications for acute nausea and vomiting in the emergency department due to their primary role as prophylactic agents, as well as considerations related to cost, availability, and significant potential for drug-drug interactions.^36^
      - Emergency physicians should be on the lookout for patients receiving highly emetogenic chemotherapeutic regimens, including platinum-based agents (cisplatin, carboplatin, oxaliplatin), alkylating agents (cyclophosphamide), anthracyclines (doxorubicin).^39^ Many common combination regimens (such as FOLFIRINOX) carry substantial emetogenic risk since they often include multiple agents with high nausea- and vomiting-inducing potential.^39^
  Suggested resources:
  Farkas J. Nausea, emesis, and antiemetics. Internet Book of Critical Care. Published April 8, 2025. Accessed May 6, 2026. https://emcrit.org/ibcc/antiemetic/
  **Case 4:**
  Your next patient is Mr. Severs. He is a 68-year-old male presenting from home via EMS with “severe dyspnea.” Paramedics share that he is currently enrolled in hospice care, but they could not discern whether the family was planning to revoke the patient’s hospice plan of care: “They were distraught and didn’t have any advanced directive paperwork available, so we brought him here to get things ironed out.” Your emergency department is full and the charge nurse asks, “Do you want me to free up a resuscitation bay or a quiet room in the back?” Mr. Severs is too dyspneic and confused to provide any history to your team. You review his chart and find the following:
  Past Medical History
  1. Rheumatoid arthritis
  2. Interstitial lung disease (ILD), chronic respiratory failure
  3. Chronic kidney disease
  Medications
  1. Hydromorphone 1–2 mg PO/sublingual every 4 hours PRN pain/dyspnea
  2. Ondansetron 8 mg PO every 8 hours PRN nausea
  3. Haloperidol 2 mg PO/sublingual every 8 hours PRN restlessness/nausea
  4. Lorazepam 1–2 mg PO/sublingual every 4 hours PRN anxiety/dyspnea
  His partner arrives with their two adult children. All three are tearful and anxious. They collectively share the following history:
  “Two weeks ago, we met with Dad’s lung doctor and kidney doctor. He had just received word that he is not a candidate for a lung transplant, something he was working toward for years. We met with the hospice team five days ago and started having visits with the team shortly after. Sure, he’s been a little more short of breath. Our home has been a large open house, with visitors coming and going all day. Today was much different. He was too drowsy and too weak to get out of bed. He told us today that his brother and sister had “stopped by”– they are no longer alive. This afternoon, he was too short of breath to even swallow medications. We called the hospice nurse, but we were told it would be an hour before they could get to the house, so we called the local paramedics. They know Dad so well and always know what to do. Something isn’t right – he had a great day yesterday! He wasn’t supposed to go downhill so fast. Please help him, Doc.”
  In Triage:
  - Temperature (T) 96.8°F
  - Heart rate (HR) 126 beats/min
  - Blood pressure (BP) 110/50 mmHg
  - Respiratory rate (RR) 32/min
  - Oxygen saturation (O_2_sat) 86% on baseline 6L nasal cannula
  Physical exam: The patient is drowsy, intermittently restless, turning frequently in the bed. His lung exam demonstrates moderate respiratory distress and rhonchi in all lung fields. He has facial grimacing and accessory muscle use. His abdomen is soft and his extremities are cool, cyanotic, and mottled in appearance. He is unable to answer questions or follow commands.
  You spend a minute confirming the patient’s goals of care with his partner/health care power of attorney, including verifying the activity of a recently signed advanced directive and Do-Not-Resuscitate order in his electronic medical record. Your assessment is that the patient is nearing end-of-life. You reassure the patient and his family that the medical team is here to support them and alleviate his symptoms of breathlessness and anxiety.
  His partner turns to you and asks, “How much time does he have, Doc?”
  **Question Prompts:**
  1. How would you estimate and communicate prognosis to Mr. Sever’s family?
    Estimating prognosis nearing end-of-life is difficult, especially in the ED where providers are relying on medical records, patient/family history, and clinical exam findings to guide discussions. There are currently no clinical calculators to easily predict survival in the final hours and days of life. Clinical clues in this patient can help to guide prognostic estimates. If the family is requesting you to predict when death will occur, it is advised to communicate your best estimate in a range. Example: minutes to hours, hours to days, days to weeks.^24^,^40^ Clinically this patient has evidence of respiratory failure, poor perfusion, and terminal delirium. While estimating prognosis is subjective, one may best put this patient’s prognosis in the hours-to-days range.
    Discussion: If a family asks for a prognosis in the end-of-life or actively dying patient, it is helpful to inquire why they are asking. Many times, this question is in reference to something specific: they want to be present at the time of death, they are trying to prepare for religious or cultural ceremonies or sacraments, they are making practical arrangements for others to visit or simply managing anticipatory grief.^40^ While estimating prognosis is difficult, ED physicians can often provide specific information that may help families adjust or expedite planning if needed. Example: “Unfortunately, while I can’t predict timelines with certainty, I would suggest that any family that wants to see him should come now.”
  2. What contributes to dyspnea at end-of-life?
    - Pneumonia^41^
    - Anemia^41^
    - Pleural effusion^41^
    - Pulmonary embolism^41^
    - Exacerbation of underlying lung disease (COPD, ILD)^41^
    - Upper or lower airway obstruction (secretions, mass)^41^
    - Anxiety^41^
    - Abdominal causes (ascites, small bowel obstruction)^41^
    - Fever^41^
  3. What non-pharmacologic and pharmacologic therapies can help with dyspnea at end-of-life?
    - Non-pharmacologic –
      - Positional changes – reposition the head to assist with ventilation, move pressure off joints that may be painful^41^–^43^
      - Bedside fan/open window – cool airflow can modulate the central perception of dyspnea by stimulating trigeminal nerve receptors^41^–^43^
      - Humidified air – helps to soften thick oropharyngeal secretions^41^–^43^
      - Quiet room – overstimulation can worsen delirium in some cases^41^–^43^
  4. Pharmacologic –
    - Oxygen – consider discontinuing unless it appears to provide symptom relief or comfort, often high flow rates and restrictive masks can contribute to delirium. Thoughtful discussions with families and other ED staff surrounding removal of oxygen at end of life may mitigate distress.^41^–^43^
    - Opioids (IV, SQ, IM, sublingual, oral liquid or tablet) – first line is morphine (caution in patients with known renal failure), hydromorphone fentanyl, or oxycodone. The most commonly used routes are IV or SQ while in hospital setting, oral and sublingual routes in the home setting. Dose and frequency will depend on the patient’s prior opioid tolerance and severity of symptoms. If high doses or increased dose frequency are required, an infusion may be needed (suggest consultation with palliative or hospice physician). In general, infusions are reserved for severe cases and are rarely appropriate as a first line treatment in the ED.^41^–^43^
    - Benzodiazepines (IV, SQ, IM, sublingual, oral liquid) – first line is lorazepam, best used when there is an anxiety component to dyspnea.^41^–^43^
    - Antipsychotics (IV, SQ, IM, sublingual, oral liquid) – can also be used if anxiolysis is needed; first line is haloperidol (except in patients with Parkinson’s Disease where quetiapine is preferred).^41^–^43^
7. After two doses of medication, the patient’s respiratory rate, restlessness and tachycardia have moderately improved. Your nurse asks how often he can receive medications and what symptoms to watch for. What is the utility of using an assessment tool for respiratory distress/dyspnea nearing end-of-life?
  Dyspnea at end-of-life is under-detected and under-treated because often medical professionals and patients perceive dyspnea differently.^42^ Also, many patients nearing end-of-life are less responsive and less-communicative, creating assessment challenges.^42^
8. What is the Respiratory Distress Observation Scale (RDOS) and how is it used?
  The RDOS is a tool used for evaluating respiratory distress in patients that are unable to self-report dyspnea. It’s a numeric scale assigned by a medical professional (RN, respiratory therapist, physician) that assesses for heart rate, respiratory rate, restlessness, paradoxical breathing, accessory muscle use, grunting at end of expiration, nasal flaring and facial expression of fear. A RDOS score of 3 or more indicates respiratory distress and suggests the addition of non-pharmacologic or pharmacologic interventions are necessary to control symptoms. RDOS can be measured at any frequency, sometimes hourly or more in highly symptomatic patients, to measure efficacy of treatment. This tool is very helpful because it provides a clinical nudge that it is time to medicate (often with an opioid) based on clinical cues. This is especially important for ED staff or providers who may feel uncomfortable caring for actively dying patients, or those who are worried they are overmedicating or contributing to or hastening the patient’s death. 42,44
  Discussion point: The Principle of Double Effect (or Doctrine of Double Effect) is a guiding bioethics principle that surrounds use of medications for symptom management at end-of-life.^45^ Academically, it can be summarized as such: if one is working to achieve something morally good (such as treating a patient’s pain and dyspnea at end-of-life) and that act has a bad side-effect (contributing to respiratory depression), it is ethical to proceed, provided the side-effect wasn’t the intended result.^45^ This principle oversees the reason ED physicians use intentional, as-needed dosing of comfort medications and refrain from the practice of extreme dosing and infusions, except in rare circumstances.
  **Table:** Respiratory Distress Observation Scale (modified by authors):^44^
  Variable	0 Points	1 Point	2 Points
  **Heart rate**	<90 bpm	90–109 bpm	≥110 bpm
  **Respiratory rate**	<19 breaths	19–30 breaths	>30 breaths
  **Restlessness**	No	Occasional	Frequent
  **Paradoxical breathing**	No		Yes
  **Accessory muscle use**	No	Slight	Pronounced
  **End-expiratory grunting**	No		Yes
  **Nasal flaring**	No		Yes
  **Look of fear**	No		Yes
  Instructions for Use:
  - Count respiratory and heart rates for one minute
  - RDOS score <3 indicates respiratory comfort
  - RDOS score ≥3 indicates need for palliation
  - Higher RDOS scores signify a worsening condition

Suggested resources for learners:’

Magidson PD, Khoujah D, Anderson RS. Palliative care in the emergency department: Simplified symptomatic management. *J Geriatr Emerg Med*. 2021; 3(2).

Murphy C, Simonis J. Assessment Tools for Dyspnea and Respiratory Distress at the End of Life. Palliative Care Network of Wisconsin. Published November 29, 2022. Accessed April 19, 2026. https://www.mypcnow.org/fast-fact/assessment-tools-for-dyspnea-and-respiratory-distressat-the-end-of-life/

Zhuang Q, Yang GM, Neo SH, Cheung YB. Validity, reliability, and diagnostic accuracy of the Respiratory Distress Observation Scale for assessment of dyspnea in adult palliative care patients. *J Pain Symptom Manag*. 2019;57(2):304–310. doi:10.1016/j.jpainsymman.2018.10.506

Xue QL. The frailty syndrome: Definition and natural history. *Clin Geriatr Med*. 2011;27(1):1–15. doi:10.1016/j.cger.2010.08.009

Selman K, Shenvi C. What’s in a name? Understanding failure to thrive and frailty in the emergency department. *J Geriatr Emerg Med*. 2022; 1(3).

Wolf LA, Lo AX, Serina P, et al. Frailty assessment tools in the emergency department: A geriatric emergency department guidelines 2.0 scoping review. *J Am Coll Emerg Physicians Open*. Published 2023 Dec 29. doi:10.1002/emp2.130842023;5(1):e13084

Fehlmann CA, Nickel CH, Cino E, Al-Najjar Z, Langlois N, Eagles D. Frailty assessment in emergency medicine using the Clinical Frailty Scale: a scoping review. *Intern Emerg Med*. 2022;17(8):2407–2418. doi:10.1007/s11739-022-03042-5

Martin FC, Ranhoff AH. Frailty and sarcopenia. 2020 Aug 21. In: Falaschi P, Marsh D, eds. *Orthogeriatrics: The Management of Older Patients with Fragility Fractures* [Internet]. 2nd edition. Cham (CH): Springer; 2021. Chapter 4. Available from: https://www.ncbi.nlm.nih.gov/books/NBK565582/

Shoenberger J, Lamba S, Goett R, et al. Development of hospice and palliative medicine knowledge and skills for emergency medicine residents: Using the Accreditation Council for Graduate Medical Education Milestone Framework. *AEM Educ Train*. 2018;2(2):130–145. Published 2018 Mar 22. doi:10.1002/aet2.10088

Smyth J, Dollard J, Visvanathan R, Archibald M. Perspectives on frailty among emergency physicians: A qualitative descriptive study. *Cureus*. 2024;16(12):e75670. Published 2024 Dec 13. doi:10.7759/cureus.75670

Otero J, Lenihan DJ. The “normothermic” Osborn wave induced by severe hypercalcemia. *Tex Heart Inst J*. 2000;27(3):316–317.

Siegel M, Bigelow S. Palliative care symptom management in the emergency department: The ABC's of symptom management for the emergency physician. *J Emerg Med*. 2018;54(1):25–32. doi:10.1016/j.jemermed.2017.08.004

Iserson KV. Withholding and withdrawing medical treatment: an emergency medicine perspective. *Ann Emerg Med*. 1996;28(1):51–54. doi:10.1016/s0196-0644(96)70139-7

## Appendix B Small Group Application Exercise for Learners

### Physical Frailty, Pallative and End-of-Life Care Case Studies

**Case 1:**

Mr. Michaels is an 84-year-old male presenting from home with a chief complaint of “fall.” He is moved from the EMS cot to your last monitored ED room. His daughter Jane rushes to his bedside, insistent that she provide his history, stating, “he is forgetful.” He is pleasant but overwhelmed by all the commotion in the room. He denies any pain, shortness of breath, headache, neck or back pain, nausea or vomiting. He cannot recall events that occurred prior to arrival. He only asks for his glasses, which seem to be missing. Jane fills in the details leading to the ED presentation:

“I was getting him up for his morning routine of breakfast and coffee. He went to pivot from the bed to his walker, when he must have lost his balance and fell, striking his head against the nightstand. I tried multiple times to get him up, but he was just too weak. I called the paramedics, and once they saw the bruise on his head and the wounds on his arms, they urged us to come in to be seen. This is his third visit to the ED in the last six weeks despite a recent hospital and rehabilitation stay. I moved him to stay with me in our spare room so I can look after him. We are doing everything the physical therapist told us, and he is still falling and barely eating. I doubt he is sleeping - neither of us are. He is restless and wants to roam the house all night. I’m not sure what more I can do. I am exhausted.”

Past Medical History

1. Parkinson’s disease
2. Benign prostatic hypertrophy
3. Vision and hearing impairment
4. Diet-controlled type 2 diabetes mellitus
5. Hypertension
6. Hyperlipidemia

Medications

1. Carbidopa-levodopa 25–100 mg PO three times daily
2. Quetiapine 50 mg PO at bedtime
3. Tamsulosin 0.4 mg PO daily
4. Atorvastatin 10 mg PO daily
5. Aspirin 81 mg PO daily

At Triage:

- Temperature (T) 98.0°F
- Heart rate (HR) 62 beats/min
- Blood pressure (BP) 106/78 mmHg
- Respiratory rate (RR) 18/min
- Oxygen saturation (O_2_sat) 97% on room air

Physical exam: The patient is alert and oriented to self and place and very cachectic; however, he appears to be in no distress. He has an abrasion to his left frontal scalp and multiple new skin tears to his upper and lower extremities. He also has a stage 1 sacral decubitus ulcer, new from the last ED visit 1 week ago. His chest, abdomen, back and neck are atraumatic. He has slight cogwheel rigidity and a resting tremor.

**Question Prompts:**

1. List the four global end-of-life trajectories, their characteristics, and include two ED patient examples of each.
2. What is physical frailty, and how prevalent is it among emergency department patients?
3. What indicators (functional changes, symptoms) suggest that a patient is nearing end-of-life (final months of life)?
4. What are the potential reversible causes of Mr. Michael’s functional decline?
5. What resources may you have in the emergency department to assist Mr. Michaels and his daughter?
6. What biases or patient history features might influence how clinicians perceive or treat patients like Mr. Michaels?

Suggested resources for learners:

Bukovitz, BJ. Top 7 things to know about frailty in the emergency department. *J Geriatr Emerg Med.* 2024; 4(5). doi:https://doi.org/10.17294/2694-4715.1102

Barker PC, Scherer JS. Illness trajectories: description and clinical use. Palliative Care Network of Wisconsin. Published February 29, 2024. Accessed April 21, 2026. https://www.mypcnow.org/fast-fact/illness-trajectories-description-and-clinical-use/

**Case 2:**

Mrs. Green is a 50-year-old female presenting with progressively worsening fatigue, nausea, severe thirst and diffuse osseous pain since receiving her last session of palliative chemotherapy four weeks ago. She also describes having severe constipation (last bowel movement was “about a week ago”), a labile and irritable mood, anorexia, and headaches, but denies fever, falls/trauma, or ill contacts.

Past Medical History

1. Breast adenocarcinoma, metastatic to the lung, liver, bone
2. Peptic ulcer disease

Medications

1. Parenteral chemotherapy q2 weeks (recently discontinued due to disease progression)
2. Omeprazole 40 mg PO daily
3. Ondansetron 8 mg PO every 8 hours PRN nausea
4. Oxycodone 5–10 mg PO every 6 hours PRN pain
5. Dexamethasone 2 mg PO twice daily
6. Antacids PRN

In Triage:

- Temperature (T) 98.8°F
- Heart rate (HR) 114 beats/min
- Blood pressure (BP) 102/67 mmHg
- Respiratory rate (RR) 22/min
- Oxygen saturation (O_2_sat) 96% on room air

Physical exam: The patient is awake and alert, answering questions and following commands. Her mucous membranes are dry. Her HR is elevated without audible murmurs. She has mild abdominal tenderness diffusely. Palpation of her extremities, especially her hips and lower legs cause her to grimace in pain. There are no wounds or rashes.

**Question Prompts:**

1. What is your differential diagnosis?
2. What ED testing would you recommend? What do expect these diagnostic studies to reveal?
3. Describe the pathophysiology of this patient’s condition as well as other etiologies of this condition.
4. What are the classic presenting symptoms and physical exam findings of this condition?
5. What urgent treatments are indicated in the emergency department for this patient?
6. What is an appropriate disposition for this patient?
7. What is the prognosis for this condition?
8. You read in the electronic medical record that the patient’s oncology team is no longer recommending palliative chemotherapy and recently offered to connect her with a local hospice agency. Is Mrs. Green eligible to invoke the Medicare Hospice Benefit?
9. What advice would you give her at this point in her illness? How do you describe hospice care to your ED patients?

Suggested resources for learners:

Farkas J. Nausea, emesis, and antiemetics. Internet Book of Critical Care. Published April 8, 2025. Accessed May 6, 2026. https://emcrit.org/ibcc/antiemetic/

Burns E, Buttner R. Hypercalcaemia. Life in the Fast Lane. Published October 8, 2024. Accessed April 19, 2026. https://litfl.com/hypercalcaemia-ecg-library/

Siddiqui F, Weissman D. Hypercalcemia of malignancy. Palliative Care Network of Wisconsin. Published July 9, 2024. Accessed April 19, 2026. https://www.mypcnow.org/fast-fact/hypercalcemia-of-malignancy/

Lamba S, Quest TE, Weissman DE. Initiating a hospice referral from the emergency department. Palliative Care Network of Wisconsin. Published April 25, 2024. Accessed April 19, 2026. https://www.mypcnow.org/fast-fact/initiating-a-hospice-referral-from-the-emergency-department

**Case 3:**

Mr. Jones is a 39-year-old male presenting with persistent non-bloody emesis for the past three days after his chemotherapy infusion (FOLFIRINOX). He notes that for the last 24 hours, despite taking in small sips of oral fluids and ondansetron (8mg PO every 6 hours), everything he eats or drinks results in emesis. Just in the past few hours, he has been experiencing acute worsening of his cancer-related epigastric abdominal pain and new lightheadedness with standing. He denies fever, chills, dyspnea or chest pain. His last bowel movement was a few hours ago and consisted of diarrhea, which is typical for him in the days following his infusions. The last time this happened he required a prolonged hospitalization.

Past Medical History

1. Metastatic pancreatic adenocarcinoma
2. GERD
3. Opioid use disorder in recovery

Medications

4. Omeprazole 20mg PO daily
5. Olanzapine 10mg PO QHS
6. Methadone 80mg PO daily
7. Oxycodone IR PO 10–20mg every 4 hours PRN
8. Multivitamin
9. Pancreatic Enzymes

Allergies

None

Vital Signs:

- Temperature (T) 37.7°C
- Blood Pressure (BP) 101/65 mmHg
- Heart Rate (HR) 114 beats/min
- Respiratory Rate (RR) 20/min
- Oxygen Saturation (O_2_sat) 95% on room air
- Weight 60kg

Physical exam: The patient has cachexia, dry mucous membranes, epigastric tenderness with minimal voluntary guarding, and no distention. The HR is elevated but regular. He has a normal mental status without focal neurological deficits, and a slow but steady gait.

Laboratory studies are pending.

The EKG is notable for sinus tachycardia with a corrected QT interval (QTc) of 480 milliseconds (ms).

**Question prompts:**

- In a general approach to emergency department patients with advanced malignancy presenting with nausea and vomiting, name at least 5 differential diagnoses:
- What components of the physical exam are the most pertinent to explore in this population?
- What laboratory and/or imaging studies are appropriate to order:
- What are the difficulties in treating this patient’s nausea?
- Fill out the following table:
  **Table:**
  Antiemetics					
  Drug	Dose	Receptor	QT	Side effects	Comments
  Ondansetron					
  Droperidol*					
  Haloperidol Lactate*					
  Prochlorperazine*					
  Promethazine*					
  Olanzapine*					
  Metoclopramide*					
  Diphenhydramine					
  Lorazepam					
  Dexamethasone					
  Isopropyl alcohol					
- What would be your next step in treating this patient’s nausea and why?

Suggested resources:

Farkas J. Nausea, emesis, and antiemetics. Internet Book of Critical Care. Published April 8, 2025. Accessed May 6, 2026. https://emcrit.org/ibcc/antiemetic/

**Case 4:**

Your next patient is Mr. Severs. He is a 68-year-old male presenting from home via EMS with “severe dyspnea.” Paramedics share that he is currently enrolled in hospice care, but they could not discern whether the family was planning to revoke the patient’s hospice plan of care. “They were distraught and didn’t have any advanced directive paperwork available, so we brought him here to get things ironed out.” Your emergency department is full and the charge nurse asks, “Do you want me to free up a resuscitation bay or a quiet room in the back?” Mr. Severs is too dyspneic and confused to provide any history to your team. You review his chart and find the following:

Past Medical History

1. Rheumatoid arthritis
2. Interstitial lung disease, chronic respiratory failure
3. Chronic kidney disease

Medications

1. Hydromorphone 1–2 mg PO/sublingual every 4 hours PRN pain/dyspnea
2. Ondansetron 8 mg PO every 8 hours PRN nausea
3. Haloperidol 2 mg PO/sublingual every 8 hours PRN restlessness/nausea
4. Lorazepam 1–2 mg PO/sublingual every 4 hours PRN anxiety/dyspnea

His partner arrives with their two adult children. All three are tearful and anxious. They collectively share the following history:

“Two weeks ago, we met with Dad’s lung doctor and kidney doctor. He had just received word that he is not a candidate for a lung transplant, something he was working toward for years. We met with the hospice team five days ago and started having visits with the team shortly after. Sure, he’s been a little more short of breath. Our home has been a large open house, with visitors coming and going all day. Today was much different. He was too drowsy and too weak to get out of bed. He told us today that his brother and sister had “stopped by” – they are no longer alive. This afternoon, he was too short of breath to even swallow medications. We called the hospice nurse, but we were told it would be an hour before they could get to the house, so we called the local paramedics. They know Dad so well and always know what to do. Something isn’t right – he had a great day yesterday! He wasn’t supposed to go downhill so fast. Please help him, Doc.”

In Triage:

- Temperature (T) 96.8°F
- Heart rate (HR) 126 beats/min
- Blood pressure (BP) 110/50 mmHg
- Respiratory rate (RR) 32/min
- Oxygen saturation (O_2_sat) 86% on baseline 6L nasal cannula

Physical exam: The patient is drowsy, intermittently restless, turning frequently in the bed. His lung exam demonstrates moderate respiratory distress and rhonchi in all lung fields. He has facial grimacing and accessory muscle use. His abdomen is soft and his extremities are cool, cyanotic and mottled in appearance.

He is unable to answer questions or follow commands.

You spend a minute confirming the patient’s goals of care with his partner/health care power of attorney, including verifying the activity of a recently signed advanced directive and Do-Not- Resuscitate order in his electronic medical record. Your assessment is that the patient is nearing end-of-life. You reassure the patient and his family that the medical team is here to support them and alleviate his symptoms of breathlessness and anxiety.

His partner turns to you and asks, “How much time does he have, Doc?”

**Question Prompts:**

1. How would you estimate and communicate prognosis to Mr. Sever’s family?
2. What contributes to dyspnea at end-of-life?
3. What non-pharmacologic and pharmacologic therapies can help with dyspnea at end-of-life?
4. After two doses of medication, the patient’s respiratory rate, restlessness and tachycardia have moderately improved. Your nurse asks how often he can receive medications and what symptoms to watch for. What is the utility of using an assessment tool for respiratory distress/dyspnea nearing end-of-life?
5. What is the Respiratory Distress Observation Scale (RDOS) and how is it used?

Suggested resources for learners:

Magidson PD, Khoujah D, Anderson RS. Palliative care in the emergency department: Simplified symptomatic management. *J Geriatr Emerg Med*. 2021; 3(2).

Murphy C, Simonis J. Assessment Tools for Dyspnea and Respiratory Distress at the End of Life. Palliative Care Network of Wisconsin. Published November 29, 2022. Accessed April 19, 2026. https://www.mypcnow.org/fast-fact/assessment-tools-for-dyspnea-and-respiratory-distressat-the-end-of-life/

Zhuang Q, Yang GM, Neo SH, Cheung YB. Validity, reliability, and diagnostic accuracy of the Respiratory Distress Observation Scale for assessment of dyspnea in adult palliative care patients. *J Pain Symptom Manag*. 2019;57(2):304–310. doi:10.1016/j.jpainsymman.2018.10.506

Xue QL. The frailty syndrome: definition and natural history. *Clin Geriatr Med*. 2011;27(1):1–15. doi:10.1016/j.cger.2010.08.009

Selman K, Shenvi C. What’s in a name? Understanding failure to thrive and frailty in the emergency department. *J Geriatr Emerg Med*. 2022; 1(3).

Wolf LA, Lo AX, Serina P, et al. Frailty assessment tools in the emergency department: A geriatric emergency department guidelines 2.0 scoping review. *J Am Coll Emerg Physicians Open*. 2023;5(1):e13084. Published 2023 Dec 29. doi:10.1002/emp2.13084

Fehlmann CA, Nickel CH, Cino E, Al-Najjar Z, Langlois N, Eagles D. Frailty assessment in emergency medicine using the Clinical Frailty Scale: a scoping review. *Intern Emerg Med*. 2022;17(8):2407–2418. doi:10.1007/s11739-022-03042-5

Martin FC, Ranhoff AH. Frailty and sarcopenia. 2020 Aug 21. In: Falaschi P, Marsh D, eds. *Orthogeriatrics: The Management of Older Patients with Fragility Fractures* [Internet]. 2nd ed. Cham (CH): Springer; 2021. Chapter 4. Available from: https://www.ncbi.nlm.nih.gov/books/NBK565582/

Shoenberger J, Lamba S, Goett R, et al. Development of hospice and palliative medicine knowledge and skills for emergency medicine residents: Using the Accreditation Council for Graduate Medical Education Milestone Framework. *AEM Educ Train*. 2018;2(2):130–145. Published 2018 Mar 22. doi:10.1002/aet2.10088

Smyth J, Dollard J, Visvanathan R, Archibald M. Perspectives on frailty among emergency physicians: A qualitative descriptive study. *Cureus*. 2024;16(12):e75670. Published 2024 Dec 13. doi:10.7759/cureus.75670

Otero J, Lenihan DJ. The “normothermic” Osborn wave induced by severe hypercalcemia. *Tex Heart Inst J*. 2000;27(3):316–317.

Siegel M, Bigelow S. Palliative care symptom management in the emergency department: The ABC's of symptom management for the emergency physician. *J Emerg Med*. 2018;54(1):25–32. doi:10.1016/j.jemermed.2017.08.004

### Physical Frailty, Pallative and End-of-Life Care Pearls

1. There are 4 end-of-life trajectories – terminal illness, organ failure, frailty, and sudden death.^21^
2. Physical frailty is defined as three of the five following: slow walking speed, fatigue, low physical activity, weight loss, weakness.^1^ It is associated with dysregulation of multiple physiologic stress responses including the immune, endocrine, inflammatory and metabolic systems, rendering patients to be at high risk for falls, depression, incontinence, disability and cognitive impairment.^1^ This is well differentiated from the pediatric term, “failure to thrive.”^22^
3. Developing a differential diagnosis and management plan for patients with life limiting illness or nearing end-of-life (falls in those with frailty, weakness and osseous pain in malignant hypercalcemia, chemotherapy-induced nausea, and terminal dyspnea) involves identifying reversible causes and clear communication regarding the patient’s treatment goals.^23^
4. When asked, it’s best to communicate prognosis using practical timeframes (hours to days, days to weeks, weeks to months, months to years).^24^
5. Often the role of an emergency physician is to connect patients nearing end-of-life with hospital or community resources such as a local hospice agency, geriatric outpatient providers, palliative care teams, social work, care management or online supportive resources. 3,23
